# Music-Evoked Reward and Emotion: Relative Strengths and Response to Intervention of People With ASD

**DOI:** 10.3389/fncir.2019.00049

**Published:** 2019-09-18

**Authors:** Eve-Marie Quintin

**Affiliations:** Department of Educational and Counselling Psychology, McGill University, Montreal, QC, Canada

**Keywords:** autism spectrum disorder, reward, emotion, music, perception, brain development

## Abstract

This review presents research findings showing that music is a unique domain to assess perception, reward, emotion, and associated physiological reactions and neural circuitry of people with autism spectrum disorder (ASD). There is growing evidence, reported in several studies in this review article, indicating that music is a relative strength of people with ASD including musical pitch perception, musical memory, and identification of music-evoked emotions. Listening to music activates neural circuits of reward and emotion response, which are described. Research presented shows adults with ASD also activate these systems when listening to music, although there may be developmental differences in the physiological and neural response to music in childhood and adolescence alongside typical behavioral response. Nonetheless, studies reviewed lend support to the use of music therapy and education for people with ASD, specifically to improve social skills and communication. Neural correlates of response to music therapy and education are also discussed. Taken together, findings reviewed provide evidence for music as a strength-based approach for ASD to assess reward and emotion response and as a powerful tool for intervention.

## The Social Role of Music

Music-making is part of the history of the human race. In 1995, paleontologist Ivan Turk found a 43,000–82,000 year old bone with characteristic wholes, which many believe to have been a flute (Wong, 1997). This archeological evidence found in northwestern Slovenia suggests that music-making dates back at least to the times of Neanderthals (Huron, 2001). Although the meaning and use of music can vary between cultures and social context, music has a universal and unique social and cultural role (Cross, 2001). Closely related to its social role, music is a powerful medium to convey emotions (Meyer, 1956). The fact that emotions are communicated through music is one of the reasons people listen to music and why people report that music listening is rewarding (Juslin and Sloboda, 2001).

## Physiological and Neural Reward Response to Music

Listening to music is often reported as a pleasurable experience among lists of everyday experiences that people enjoy engaging in (Dubé and Le Bel, 2003). Some people even report intense euphoria when listening to emotionally intense and very pleasant music, also known as “musical chills” (Goldstein, 1980; Panksepp, 1995). Subjective ratings of musical chills are associated with a characteristic physiological response of autonomic nervous system arousal including increase in heart rate, respiration depth (Blood and Zatorre, 2001; Salimpoor et al., 2011), electromyogram signal (Blood and Zatorre, 2001), electrodermal response, and decreased temperature and blood volume pulse amplitude (Salimpoor et al., 2011). At the neural level, PET and fMRI studies showed that musical chills are associated with activity in several regions including the hypothalamus, which may point to pleasant music eliciting an autonomic response given its role in controlling heart rate and respiration (Menon and Levitin, 2005), the nucleus accumbens (NAc) and ventral tegmental area (VTA), which suggests occurrence of a reward response (Blood and Zatorre, 2001; Menon and Levitin, 2005), and of the left amygdala (Blood and Zatorre, 2001), which is associated with processing of music-evoked emotions along with the NAc and hypothalamus (Koelsch, 2014). The NAc and VTA are part of the dopaminergic reward response system for stimuli that elicit intense emotions and pleasure such as food, monetary reward, and certain psychoactive drugs (Egerton et al., 2009). A study combining [^11^C]raclopride PET and fMRI showed increase in endogenous dopamine transmission involving the right NAc during musical chills, and the right caudate nucleus in anticipation of these moments, thus confirming that listening to pleasurable music activates the dopaminergic reward system (Salimpoor et al., 2011).

## Music and Autism Spectrum Disorder (ASD)

As per the fifth edition of the *Diagnostic and Statistical Manual of Mental Disorders*, autism spectrum disorder (ASD) is defined as “persistent deficits in social communication and social interaction across multiple contexts [and] restricted, repetitive patterns of behavior, interests, or activities” (American Psychiatric Association, 2013). Knowledge of genetic risk factors associated to neurological development and to ASD is constantly improving thanks to advances in genetic techniques (Woodbury-Smith and Scherer, 2018) such as induced pluripotent stem cells derived neurons (Zaslavsky et al., 2019). At a behavioral level, there is growing interest in the strengths associated with ASD (Mottron et al., 2006). There is growing evidence that music perception and ability are one of the relative strengths of people with ASD; relative to their difficulties (Heaton, 2009). Although research on music and ASD has been flourishing since the 1990s, the idea that music is a strength within the ASD profile dates back to Kanner (1943). He described 11 children with ASD including six children with a particular musical interest or ability. In a now seminal study, Heaton et al. (1998) taught children with ASD to associate syllables or musical notes (pitches) with pictures of an animal. One week later, children with ASD and a comparison group with typical development matched for mental age remembered syllables equally well but the group of children with ASD showed more accurate memory for pitches (Heaton et al., 1998). A more recent study has extended this finding, showing that it also holds for memory of musical melodies (Stanutz et al., 2014). Further, children with ASD can easily label each pitch when several pitches are played together in a musical chord, known as chord segmentation, when they have been previously exposed to individual pitches (Heaton, 2003). People with ASD also display enhanced recognition of alterations of single pitches in a melody, even when the altered note remains in tune with the scale of the melody such that most people would not detect this type of alteration (Mottron et al., 2000). Similarly, they can distinguish between two interleaved melodies played at the same frequency, which are melodies played simultaneously but organized such that notes of one melody do not co-occur with the notes of the other melody (Bouvet et al., 2016). Enhanced pitch perception is an example of perceptual strengths of people with ASD that Mottron et al. (2014) suggest to stem from brain plasticity and reorganization triggered by genetic factors, which also lead to reduced socialization.

People with ASD also show enhanced discrimination of changes of less than a semitone, the smallest interval between two pitches in western music (Bonnel et al., 2010), and discrimination of similar sound frequencies (e.g., noticing the difference between 1,000 Hz vs. 1,030 Hz; Bonnel et al., 2003). Bhatara et al. (2013a) also found typical discrimination of pitches at 500 and 1,000 Hz, but do not at 4,000 Hz. Thus, a perceptual enhancement may be specific to detecting small pitch changes and changes at low frequencies (<4,000 Hz). It has also been suggested that enhanced auditory discrimination is present in only 20% of people with ASD (Jones et al., 2009), which could explain mixed findings in studies with small sample sizes. Further, we wonder if modifying experimental paradigms to allow more time for participants to respond would have lead to different results because people with ASD showed delayed auditory response of 11 ms as measured with MEG response of the superior temporal gyrus to sound frequencies below 1,000 Hz (Roberts et al., 2010). Such a delay may explain why adolescents with ASD are not as accurate as those with typical development at processing small gaps (in milliseconds) of silence between tones (Bhatara et al., 2013a) and why adults with ASD are less accurate than typically developing peers at judging duration of auditory events (Kargas et al., 2015). However, despite potential differences in perception of specific sound frequencies and in timing perception at the millisecond level, children and adolescents with ASD can accurately judge duration of musical events (Jones et al., 2009), perceive meter in western music (DePape et al., 2012), and are able to create and replicate musical melodies with coherent musical structure in a similar amount of time as typically developing peers (Quintin et al., 2013).

Furthermore, adolescents with ASD show as much interest in music and spend as many hours per week listening to music as typically developing peers, as revealed by both self and parent report (Bhatara et al., 2013b). Adults with ASD also report listening to music for similar reasons as typical listeners including to relax or to cheer up or feel better (Allen et al., 2009), indicating that music listening is a rewarding activity for people with ASD, and may thus activate the dopaminergic response system, in part due to its effect on emotions and mood regulation. Indeed, typical recognition of music-evoked emotions has been reported on several occasions. Children with ASD can accurately identify happy, sad (Heaton et al., 1999; Quintin et al., 2011), scary, and peaceful music (Quintin et al., 2011). They can also associate images of social scenes depicting an emotion with a corresponding musical excerpt (Heaton et al., 2008).

The connection between music and ASD lead Bergmann et al. (2015, 2016) to develop the Music-based Scale for Autism Diagnosis (MUSAD), conceived as a musical equivalent of the Autism Diagnostic Observation Schedule (ADOS-2; Lord et al., 2012). The MUSAD consists of several joint music-making activities during which social interactions and stereotyped, restricted, and repetitive behaviors can be prompted, observed, and quantified. Given that joint music making elicits nonverbal communication, the MUSAD can inform the differential diagnosis between ASD and intellectual developmental disorders with minimally verbal older adults who do not have the language skills to complete the advanced modules of the ADOS-2 and for whom the materials of the lower modules are not age appropriate (Bergmann et al., 2015, 2016).

## Physiological and Neural Response to Music and ASD

Despite relative strengths in music perception, there may be developmental differences in response to music-evoked emotions. Children and adolescents (Stephenson et al., 2016) and adults (Allen et al., 2013) with ASD can accurately recognize music-evoked emotions. However, Stephenson et al. (2016) reported that children and adolescents had lower skin conductance response than typically developing peers when listening to music, while the two groups showed similar baseline skin conductance during a visual task. In contrast, Allen et al. (2013) did not find signs of reduced skin conductance when adults with ASD listened to the same music used by Stephenson et al. (2016) as compared to listening to environmental noise or silence. Taken together, these findings may suggest that ASD is associated with a unique developmental trajectory of physiological response to music evoked-emotions perhaps affecting embodied cognition, the relevance and salience of environmental stimuli (Klin et al., 2003), and neural and behavioral development (Rosenberg et al., 2015). In Allen et al.’s (2013) study, participants with ASD listed fewer words than a comparison group of typical adults when asked to describe music-evoked emotions, which was accounted for by the presence of type II alexithymia symptoms in the ASD group. In other words, adults with ASD “feel” music-evoked emotions but explain what they feel in few words.

Neuroimaging studies can also explain the relationship between physiological and behavioral responses to music-evoked emotions. Adults with ASD show typical behavioral continuous ratings (very sad to very happy; Gebauer et al., 2014) and valence (negative to positive) and arousal (low to high energy) ratings (Caria et al., 2011) accompanied by overall typical neural processing of music including auditory and motor areas and activation of regions involved in the dopaminergic reward response system (Caria et al., 2011; Gebauer et al., 2014) to “musical chills” as described above (Blood and Zatorre, 2001; Menon and Levitin, 2005; Salimpoor et al., 2011). Specifically, contrasting activation for happy or sad music to chromatic scales (neutral stimuli) revealed activation of ventral striatum and NAc for adults with ASD, as was the case for adults in a typical comparison group (see reproduction of Figure 1 from Gebauer et al., 2014). Considering participants’ musical preference revealed that adults with ASD showed activation of the medial prefrontal cortex when listening to preferred happy music, of the VTA for preferred sad music, and of the caudate nucleus for preferred and non-preferred happy music (Caria et al., 2011). Processing of music-evoked emotions was also associated with activation of medial orbitofrontal cortex and limbic and paralimbic areas extending into the amygdala (Gebauer et al., 2014). Music thus activates typical reward and emotion processing systems of people with ASD. However, the insula, an area associated to awareness and cognitive processing of emotional states, showed both hyper- (see reproduction of Figure 2 from Gebauer et al., 2014) or hypo-activation (Caria et al., 2011). This mixed finding may be related to the fact that novel music was presented in one study (Gebauer et al., 2014) while well-known and participant selected music was used in the other (Caria et al., 2011). Alternatively, conscious awareness of music-evoked emotions may vary more for adults with ASD than without ASD.

**Figure 1:**
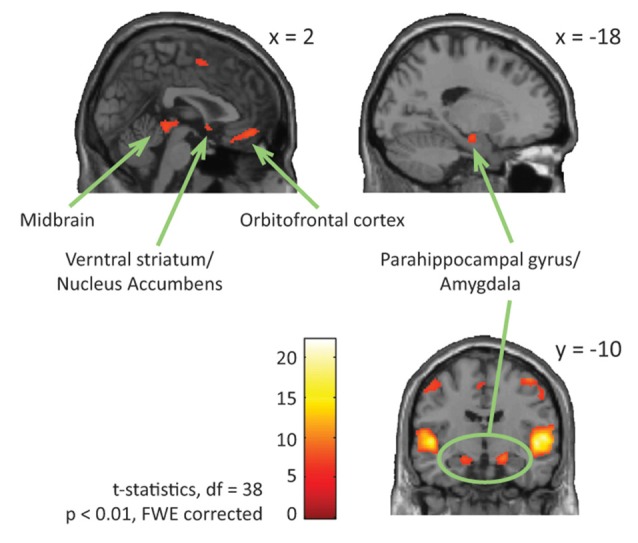
ASD and NT-main effect of emotional vs. neutral music. The figure shows brain activations in response to emotional compared to neutral music across all participants, including activations in midbrain, parahippocampal gyrus extending into amygdala, ventral striatum/nucleus accumbens, and orbitofrontal cortex. This figure is a reproduction of Figure 4 from Gebauer et al. (2014).

**Figure 2:**
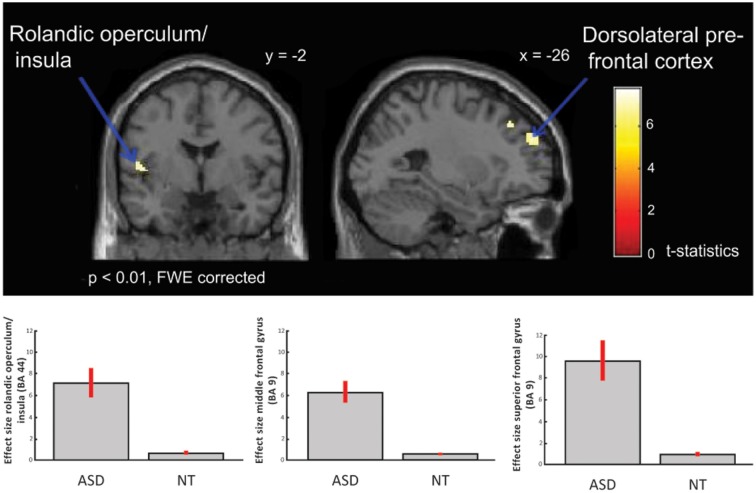
ASD > NT: group difference for happy vs. sad music (FWE p < 0.01). Individuals with ASD showed increased activation in dorsolateral prefrontal cortex, i.e., middle and superior frontal gyrus, and in insula/rolandic operculum. Box plots show mean effect size for each group in the peak voxel for each region, with 95% confidence intervals. This figure is a reproduction of Figure 3 from Gebauer et al. (2014).

Neuroimaging studies of music and ASD with children have mainly assessed neural response to songs and speech, without an emphasis on emotions. Children with ASD show typical recruitment of auditory areas when processing songs (Lai et al., 2012; Sharda et al., 2015). However, when listening to songs compared to speech, they show greater activation in left inferior frontal gyrus and posterior brain areas and increased functional connectivity between left inferior frontal gyrus and superior temporal gyrus and between frontal and posterior areas (Lai et al., 2012). Similarly, Sharda et al. (2015) found greater connectivity of the left inferior frontal gyrus with bilateral posterior temporal, right parieto-occipital, and right cerebellar regions when children with ASD listen to sung compared to spoken words. These findings suggest that although fronto-temporal circuits are involved in processing both speech and song, these circuits seem more responsive to processing songs for children with ASD perhaps due to changes in pitch occurring slower in songs than in speech (Lai et al., 2012). Listening to music and songs is thus a rewarding experience and powerful medium to communicate emotions for many people including people with ASD.

## Music Therapy and ASD and Music Education in the General Population

Given the musical strengths of children and adolescents with ASD, many families engage their child with ASD in music therapy, which can improve social outcomes (LaGasse, 2017; see Table 1). “Music therapy techniques include free and structured improvisation, singing songs and vocalization, and listening to both pre-recorded and live music” leading to nonverbal musical interactions between the participant and therapist that scaffold learning to communicate, partake in social interaction, and express emotions through music (Geretsegger et al., 2014). A review of 10 studies showed that music therapy can improve social interaction, verbal communication, initiating behavior, and social-emotional reciprocity (Geretsegger et al., 2014). However, a large randomized clinical trial including nine countries did not find effects of improvisational music therapy on social skills measured with the ADOS social affect domain and Social Responsiveness Scale (SRS; Bieleninik et al., 2017). Sharda et al. (2018) also failed to find changes in the SRS-2 associated with music therapy for ASD, but reported that music therapy improves communication and quality of life. Further, improvements in communication were associated with increased auditory-subcortical and decreased auditory-visual functional brain connectivity. Taken together, these studies show the importance of choice of outcome measures for assessing the effects of music therapy and show that it can have positive effects on social and communicative skills of children with ASD. Whether these effects are comparable to those of social skills intervention programs such as the PEERS (Laugeson et al., 2015) and JASPER (Kasari et al., 2015) programs or theatre interventions (Corbett et al., 2016) remains tobe established.

**Table 1:** Findings of studies on the effects of music therapy for autism spectrum disorder (ASD).

Reference	Main findings
Sharda et al. (2018)	Improvements in communication skills associated with resting stage auditory-subcortical and auditory-fronto-motor brain connectivity.
Mössler et al. (2019)	Improvements in social skills, communication, and language are associated with the quality of the relationship with the therapist.
Bieleninik et al. (2017)	No significant difference in improvement of ASD symptom severity.
Carpente (2016)	Improvements in self-regulation, engagement, communication.
Ghasemtabar et al. (2015)	Improvements in social skills.
LaGasse (2014)	Improvements in joint attention with peers, eye gaze towards persons; no improvements in initiation or response to communication or social withdrawal.
Thompson et al. (2014)	Improvements in social interactions in the home, quality of parent-child relationship (this is a study of family-centered music therapy); no improvements in language skills or general social responsiveness.
Gattino et al. (2011)	Improvements in nonverbal communication for autism subgroup, but no improvements in nonverbal, verbal, and social communication for all participants combined.
Hillier et al. (2012)	Improvements in self-esteem, attitude towards peers, reduced anxiety.
Kim et al. (2009)	Improvements in emotional synchrony, initiation of engagement, compliance with music therapist, and displaying joy.
Kim et al. (2008)	Improvements in joint attention behaviors, nonverbal social communication, eye contact, and turn-taking.
Boso et al. (2007)	Decrease of ASD and psychiatric symptoms.
Kern and Aldridge (2006)	Facilitation of play and interaction with peers; no improvements in social skills.
Ma et al. (2001)	Improvement in communication skills.

*This table includes peer-reviewed studies on music therapy for ASD published since 2000. This table does not include studies where music is incorporated to therapy (e.g., adding music to speech therapy or to ABA intervention)*.

Music education for ASD has not been studied as extensively as music therapy and holds promise to have positive effects given that music is one of their relative strengths. The main goal of music education is to develop musical skills, knowledge, and abilities, and often follows a set curriculum (Salvador and Pasiali, 2017). The focus is to gain musical performance skills, while music therapy targets personal development (Salvador and Pasiali, 2017). Music education in the general population is associated with improvements that transfer to enhanced non-musical skills including near transfer to working memory (Bailey and Penhune, 2010) and executive functioning (Moreno et al., 2011), that are practiced when learning to play an instrument or learning to sign, and far transfer to verbal skills (Schellenberg, 2004, 2011) and verbal memory (Ho et al., 2003; Roden et al., 2012), likely because mechanisms involved in music and speech processing overlap (Patel, 2011, 2014). As such, musical training leads to greater improvements in language skills and executive functions than visual art training (Moreno et al., 2011). Further, the use of music in speech therapies for ASD is associated with positive outcomes and increased language gains (Lim and Draper, 2011; Chenausky et al., 2016). In addition, music education of typically developing children and adolescents has been shown to result in brain plasticity, as exemplified by shorter delays in neural response to speech sounds in noise (Kraus and Strait, 2015), increased neural discrimination of small pitch changes in speech (Carpentier et al., 2016) and of spoken syllables (Kraus et al., 2014), enhanced neural correlates of executive functioning (Moreno et al., 2011), and enlargement of brain areas associated with music perception including the auditory cortex and limbic areas (Hyde et al., 2009); with studies including comparisons of music education to physical fitness (Kraus and Strait, 2015), visual arts training (Moreno et al., 2011), second language instruction (Carpentier et al., 2016), exposure to musical instruments without formal instruction (Hyde et al., 2009), and longitudinal investigations of effects of music education over time (Kraus et al., 2014). It is also worth noting that music education is related to enhanced visual-perceptual skills (Schellenberg, 2004, 2011) and that joint music-making promotes social skills (Kirschner and Tomasello, 2010; Rabinowitch and Meltzoff, 2017).

In sum, music therapy can have a positive impact on social and communication skills of children and adolescents with ASD while music education holds the potential to improve their working memory, executive functioning and language skills. Individualized interventions should include music therapy or education or their combination depending on intervention goals. The fields of music therapy and education may have much to gain from merging their approaches.

## Summary

Musical ability and interest are relative strengths of people with ASD including enhanced musical memory, enhanced or typical perception of pitches and sound frequencies, and typical perception of music-evoked emotions. When listening to music, adults with ASD show typical activation of neural circuitry associated with reward and emotion processing including NAc, VTA, ventral striatum, amygdala, medial prefrontal and orbitofrontal cortex. Adults with ASD also have a typical galvanic skin response to music-evoked emotions, but this response seems decreased in children and adolescents with ASD. In contrast, fronto-temporal circuits of children with ASD are particularly responsive to songs. There may thus be different physiological and neural developmental trajectories in response to a few aspects of music, but overall typical processing, alongside typical behavioral response to music-evoked emotions among people with ASD. Music is a powerful therapeutic and educational tool for people with ASD, with research showing improvements in social and communicative skills associated with music therapy. The growing field of music and ASD research holds promise for revisiting our understanding of social and emotional profiles of people with ASD and providing strength-based interventions.

## Author Contributions

The author confirms being the sole contributor of this work and has approved it for publication.

## Conflict of Interest Statement

The author declares that the research was conducted in the absence of any commercial or financial relationships that could be construed as a potential conflict of interest.
